# Co‑cultivation of anaerobic fungi with *Clostridium acetobutylicum* bolsters butyrate and butanol production from cellulose and lignocellulose

**DOI:** 10.1093/jimb/kuac024

**Published:** 2022-11-03

**Authors:** Jennifer L Brown, Matthew A Perisin, Candice L Swift, Marcus Benyamin, Sanchao Liu, Vasanth Singan, Yu Zhang, Emily Savage, Christa Pennacchio, Igor V Grigoriev, Michelle A O'Malley

**Affiliations:** Department of Chemical Engineering, University of California Santa Barbara, Rm 3357 Engineering II, Santa Barbara, CA 93117, USA; Biological and Biotechnology Sciences Division, DEVCOM Army Research Laboratory, 2800 Powder Mill Road, Adelphi, MD 20783, USA; Department of Chemical Engineering, University of California Santa Barbara, Rm 3357 Engineering II, Santa Barbara, CA 93117, USA; Biological and Biotechnology Sciences Division, DEVCOM Army Research Laboratory, 2800 Powder Mill Road, Adelphi, MD 20783, USA; Biological and Biotechnology Sciences Division, DEVCOM Army Research Laboratory, 2800 Powder Mill Road, Adelphi, MD 20783, USA; US Department of Energy Joint Genome Institute, Lawrence Berkeley National Laboratory, Berkeley, CA 94720, USA; US Department of Energy Joint Genome Institute, Lawrence Berkeley National Laboratory, Berkeley, CA 94720, USA; US Department of Energy Joint Genome Institute, Lawrence Berkeley National Laboratory, Berkeley, CA 94720, USA; US Department of Energy Joint Genome Institute, Lawrence Berkeley National Laboratory, Berkeley, CA 94720, USA; US Department of Energy Joint Genome Institute, Lawrence Berkeley National Laboratory, Berkeley, CA 94720, USA; Department of Plant and Microbial Biology, University of California Berkeley, Berkeley, CA 94720, USA; Department of Chemical Engineering, University of California Santa Barbara, Rm 3357 Engineering II, Santa Barbara, CA 93117, USA; Joint BioEnergy Institute, Lawrence Berkeley National Laboratory, Berkeley, CA 94608, USA

**Keywords:** Anaerobic fungi, Clostridia, RNA-Seq, Consortia, Biofuel

## Abstract

A system for co-cultivation of anaerobic fungi with anaerobic bacteria was established based on lactate cross-feeding to produce butyrate and butanol from plant biomass. Several co-culture formulations were assembled that consisted of anaerobic fungi (*Anaeromyces robustus, Neocallimastix californiae*, or *Caecomyces churrovis*) with the bacterium *Clostridium acetobutylicum.* Co-cultures were grown simultaneously (e.g., ‘one pot’), and compared to cultures where bacteria were cultured in fungal hydrolysate sequentially. Fungal hydrolysis of lignocellulose resulted in 7–11 mM amounts of glucose and xylose, as well as acetate, formate, ethanol, and lactate to support clostridial growth. Under these conditions, one-stage simultaneous co-culture of anaerobic fungi with *C. acetobutylicum* promoted the production of butyrate up to 30 mM. Alternatively, two-stage growth slightly promoted solventogenesis and elevated butanol levels (∼4–9 mM). Transcriptional regulation in the two-stage growth condition indicated that this cultivation method may decrease the time required to reach solventogenesis and induce the expression of cellulose-degrading genes in *C. acetobutylicum* due to relieved carbon-catabolite repression. Overall, this study demonstrates a proof of concept for biobutanol and bio-butyrate production from lignocellulose using an anaerobic fungal-bacterial co-culture system.

## Introduction

Synthetic microbial consortia are promising potential avenues for bio-based chemical production, including the production of biofuels from waste materials. The co-cultivation of microbes with complementary metabolic functions also eliminates the need for genetic manipulation of recalcitrant microbes and provides a division of labor between members to avoid metabolic burden (Jiang et al., 2020; Roell et al., 2019). Stable microbial consortia are found in natural environments ranging from soil microbiomes to the digestive tract of herbivores. Across these environments, consortium stability and function depend upon several factors, including functional complementation, redundancy between community members, and nutrient cross-feeding to relax metabolic burdens (Freilich et al., 2011; Peng et al., 2021; Söllinger et al., 2018).

Microbial consortia drive lignocellulose breakdown in the digestive tract of herbivores (Groussin et al., 2017; Yang & Wyman, 2008), and provide an attractive avenue for lignocellulosic biofuel production. Recently anaerobic fungi were shown to be efficient degraders of lignocellulosic biomass, eliminating the need for costly and energy-intensive pretreatment processes that produce inhibitory byproducts (Haitjema et al., 2017; Sanderson, 2011; Solomon et al., 2016). The anaerobic fungi also release excess sugars during lignocellulose breakdown (Henske et al., 2018) as well as other fermentation products such as acetate, lactate, H_2_, and ethanol. In the herbivore rumen, these fungal metabolites are understood to support the growth of other gut microbes such as archaeal methanogens to release methane (Bauchop & Mountfort, 1981; Hess et al., 2020; Theodorou et al., 1996), but they can also be used to drive growth and bioproduction in microbes that are not native to the rumen microbiome (Brown et al., 2021; Henske et al., 2018; Li et al., 2022; Swift et al., 2019). While the anaerobic fungi lack reliable genetic tools (Hooker et al., 2019), they can still be harnessed for bioproduction as members of synthetic consortia. These consortia combine the powerful carbohydrate active enzyme (CAZyme) production of anaerobic fungi with genetically tractable microbes that can cross-feed on fungal fermentation products to synthesize value-added chemicals and fuels from lignocellulose.

Here, we constructed and evaluated several synthetic microbial consortia that paired anaerobic fungi with the anaerobic bacterium *Clostridium acetobutylicum. Clostridium acetobutylicum* is not native to the rumen microbiome, and cannot digest lignocellulose, but can produce acetone, butanol, and ethanol from fermentable sugars (via ABE fermentation) (Sreekumar et al., 2015). Two key phases occur during batch fermentation of *C. acetobutylicum* (Amador-Noguez et al., 2010; Bennett & Rudolph, 1995; J. Lee et al., 2008; Walter et al., 1992; Welch et al., 1989; Wiesenborn et al., 1989). During exponential growth, *C. acetobutylicum* produces acetate and butyrate resulting in a lower culture pH (acidogenesis). These short-chain fatty acids are then reassimilated during solventogenesis, corresponding to an increase in pH, production of acetone, n-butanol, and ethanol, and initiation of bacterial sporulation (Al-Hinai et al., 2015; Paredes et al., 2005). While microbially sourced biobutanol is not currently economically competitive with petrochemical synthesis, advances in genetic engineering of *C. acetobutylicum* and improvements to downstream processes have been shown to elevate n-butanol production in *C. acetobutylicum* (S. Y. Lee et al., 2012; Xue et al., 2017).

Pairing *C. acetobutylicum* with an efficient lignocellulosic biomass degrader like an anaerobic fungus provides an economic advantage by enabling bio-based production of fuels from low-cost feedstocks (J. Wang et al., 2014). For example, the excess sugars released by anaerobic fungi during biomass breakdown (Henske et al., 2018) could potentially support the growth of *C. acetobutylicum*, which can utilize both hexose and pentose sugars but has been shown to preferentially metabolize hexose sugars over pentose sugars (Aristilde et al., 2015). Similar co-culture strategies have been previously used, where anaerobic fungus *Pecoramyces* sp. F1 and associated methanogens were able to pretreat and saccharify lignocellulosic biomass to support ethanol production by the bacterium *Z. mobilis* (Li et al., 2022). Moreover, a previous study combined *C. acetobutylicum* with undefined rumen fluid to hydrolyze pretreated agave and enhance hydrogen and butanol production (Morales-Martínez et al., 2020). Based on prior work and our current knowledge of anaerobic fungal metabolism (Henske et al., 2018; Wilken et al., 2021), we hypothesized that metabolic cross-feeding of lactate and/or succinate between anaerobic fungi and *C. acetobutylicum* would enable synergistic growth. In turn, *C. acetobutylicum*’s uptake of the lactate produced by co-cultured anaerobic fungi would relieve product inhibition experienced by the fungi in monoculture.

Here, we construct and compare two co-culture strategies to anaerobically produce butyrate and butanol from plant-derived biomass. In the first strategy, we simultaneously co-cultured several strains of anaerobic fungi with bacterium *C. acetobutylicum* on lignocellulose and measured generated fermentation products over the course of nearly 30 days. In the second strategy, fungi were initially cultivated for 22 days to release fermentable sugars and metabolic byproducts, and *C. acetobutylicum* was added directly to fungal supernatant to facilitate butyrate/butanol production. There was at least 4.5 mM more average butyrate produced in the one-stage cultivation condition versus the two-stage condition, with as much as 30 mM of butyrate being produced by *C. acetobutylicum* paired with the *N. californiae* strain after 30 days of fermentation. Alternatively, significantly more butanol was produced in all experimental conditions compared to *C. acetobutylicum* monoculture controls for long-term cultivation, with as much as 9 mM butanol being produced by *C. acetobutylicum* paired with the *N. californiae* strain in the two-stage experimental condition. While there remains significant room for optimization following these initial studies, we have shown that long-term cultivation of different strains of anaerobic fungi and *C. acetobutylicum* is a promising route for bio-butyrate and biobutanol production, and that lactate cross-feeding likely occurs between anaerobic fungi and *C. acetobutylicum*, which bolsters butyrate production by *C. acetobutylicum* in the co-cultivation condition.

## Materials and Methods

### Routine Microbial Cultivation

The anaerobic gut fungal strains, *Neocallimastix californiae* (Haitjema et al., 2017; Solomon et al., 2016), *Anaeromyces robustus* (Haitjema et al., 2017; Solomon et al., 2016), and *Caecomyces churrovis* (Brown et al., 2021; Henske et al., 2017), were isolated as described previously. The fungal cultures were anaerobically cultivated at 39°C in Hungate tubes with 100% CO_2_ headspace, 10 ml of Medium C (MC) or Minimal Medium 2 (M2), and 0.5 g reed canary grass as the carbon source as described previously (Teunissin et al., 1991; Theodorou et al., 2005). The reed canary grass was provided by the US Department of Agriculture, Agricultural Research Service, US Dairy Forage Research Center, and was milled in a Model 4 Wiley Mill (Thomas Scientific) using a 4 mm screen size (courtesy of P. J. Weimer). Growth of anaerobic fungi was monitored via a pressure transducer method used to measure the accumulation of fermentation gas in the headspace of the culture tubes (Theodorou et al., 1995).

*Clostridium acetobutylicum* ATCC 824 obtained from the American Type Culture Collection (ATCC) was anaerobically maintained as a spore suspension stock in potato glucose medium (PGM) (Al-Shorgani et al., 2014) containing 150 g/l potato (shredded, boiled for 1 hr, and filtered through cheesecloth), 1% glucose, 30 mM CaCO_3_, and 4 mM (NH_4_)_2_SO_4_. *Clostridium acetobutylicum* spore stock was revived by heating 1 ml at 80°C for 10 min on a heat block before adding heat-shocked spore stock to a 15 ml Falcon tube containing 8 ml of clostridium growth medium (CGM) (Wiesenborn et al., 1989) and 0.5 g/ml glucose solution. This culture was subsequently used to make a 1:10, 1:100, and 1:1000 dilution in three additional Falcon tubes prepared with 8 ml CGM and 0.5 g/l glucose solution. The spore stock dilution cultures were grown at 39°C in an anaerobic chamber for ∼24 hr until one of the dilutions reached an OD600 of 0.8–1, at which time it could be used to inoculate a seed culture for the experiments.

### Short-Term Simultaneous (One-Stage) Co-Cultivation

An *A. robustus* seed culture was grown by adding 2 ml of an *A. robustus* 10 ml culture grown for 3 days in a Hungate tube in Medium C (Theodorou et al., 2005) with a reed canary grass as a substrate to an autoclaved PYREX 250 ml Delong Shaker Flask with Extra-Deep Baffles containing 0.8 g filter paper and 38 ml of anaerobic undefined Medium B at 39°C (Lowe et al., 1985). This culture and all other seed and experimental cultures were grown unshaken in a modified version of Medium B either defined (no yeast extract or Bacto Casitone added) or undefined (yeast extract and Bacto Casitone added) with only 1 g/l Na_2_CO_3_ in order to lower the pH of the Medium B, making it suitable to cultivate both microbes used in the study (Lowe et al., 1985). The Whatman filter paper was cut into ∼0.5 inch strips for all cultures. This culture was grown in undefined Medium B for 3 days at 39°C in an AS-580 gloveless anaerobic chamber (Anaerobe Systems, Morgan Hill, CA, USA) before being used to inoculate experimental cultures. Two ml of the *A. robustus* seed culture was used to inoculate four PYREX 250 ml Delong Shaker Flasks with Extra-Deep Baffles containing 0.8 g filter paper, 34 ml defined Medium B, and 2 ml 10 wt/vol% maltodextrin solution at 39°C to form the experimental co-cultures after the *A. robustus* has grown for 24 hr to establish the fungal population since *A. robustus* grows slower than *C. acetobutylicum*. At the same time, four additional *A. robustus* cultures were inoculated with 2 ml of *A. robustus* seed culture in autoclaved PYREX 250 ml Delong Shaker Flasks with Extra-Deep Baffles with 36 ml anaerobic MB, 2 ml maltodextrin, and 0.8 g filter paper at 39°C to serve as *A. robustus* monoculture controls grown in the anaerobic chamber at 39°C.

*Clostridium acetobutylicum* spore stock was revived by heating 1 ml at 80°C for 10 min on a heat block before adding heat-shocked spore stock to a 15 ml Falcon tube containing 8 ml of CGM and 0.5 g/ml glucose solution. This culture was subsequently used to make a 1:10, 1:100, and 1:1000 dilution in three additional Falcon tubes prepared with 8 ml CGM and 0.5 g/l glucose solution. The spore stock dilution cultures were grown at 39°C in an anaerobic chamber for ∼24 hr until one of the dilutions reached an OD600 of 0.8–1, at which time it could be used to inoculate a seed culture. The 1:1000 dilution reached OD600 of 1.386 at ∼24 hr, therefore 2 ml of that culture was used to inoculate a *C. acetobutylicum* seed culture in an autoclaved yeast shaker flask filled with 36 ml anaerobic undefined Medium B and 2 ml of 10 wt/vol% maltodextrin solution at 39°C. This *C. acetobutylicum* seed culture was grown for 24 hr at 39°C in an anaerobic chamber before being used to inoculate experimental cultures. After ∼24 hr, the culture had reached an OD600 of 0.973. Four *C. acetobutylicum* monoculture controls were inoculated with 2 ml of the *C. acetobutylicum* seed culture in autoclaved yeast shaker flasks containing 36 ml defined Medium B and 2 ml 10 wt/vol% maltodextrin at 39°C. After the flasks inoculated with the 2 ml of the *A. robustus* seed culture had grown in the anaerobic chamber at 39°C for ∼24 hr, 2 ml of the *C. acetobutylicum* seed culture was added to each flask to form the co-cultures.

All cultures were covered with parafilm until the *C. acetobutylicum* inoculum was added to prevent evaporation of the liquid. A sample of 250 μl of Medium B from all four replicates of the *C. acetobutylicum* monocultures, *C. acetobutylicum* and *A. robustus* co-cultures, and *A. robustus* monocultures was taken before inoculation and placed in 1.5 ml Eppendorf^™^ tubes and stored at −80°C for later HPLC analysis. After inoculation, a 250 μl sample of each culture supernatant was taken at 2-hr intervals for a 12-hr period and stored in 1.5 ml Eppendorf^™^ tubes at −80°C for later analysis. The culture was then allowed to grow without sampling for 10 hr overnight, then 250 μl samples for HPLC analysis were again collected and stored at −80°C for later HPLC analysis at 4-hr intervals for 12 hr, with a final reading taken 2 hr later. The cultures were then allowed to grow undisturbed for another 12 hr before collecting a final 250 μl HPLC sample from each culture before harvesting the cultures for RNA extraction. The OD600 measurement of each culture was taken before harvest and ranged from 0.07–0.119.

### Short-Term Sequential (Two-Stage) Co-Cultivation

An *A. robustus* seed culture was grown by adding 1 ml of an *A. robustus* 10 ml culture grown in a Hungate tube in Medium C with a reed canary grass substrate to an autoclaved yeast shaker flask with 0.8 g filter paper, 37 ml of anaerobic defined Medium B, and 2 ml of 10 wt/vol% sterile filtered maltodextrin solution at 39°C in an AS-580 gloveless anaerobic chamber (Anaerobe Systems, Morgan Hill, CA, USA). The culture grew undisturbed at 39°C in the anaerobic chamber for 3 days. After 3 days, the culture was checked for signs of growth. Bubbling was observed in the culture, indicating that it grew successfully. Four autoclaved yeast shaker flasks containing 0.8 g filter paper, 37 ml defined anaerobic Medium B, and 2 ml of 10 wt/vol% maltodextrin were inoculated with 1 ml of the *A. robustus* seed culture. The flasks were then placed in the 39°C incubator in the anaerobic chamber to grow for eight days. After eight days, the fungal supernatant was sterile filtered and 35 ml was transferred to four autoclaved yeast shaker flasks containing 0.8 g filter paper and inoculated with 1 ml of the *C. acetobutylicum* seed culture once the seed culture had reached an OD600 of 1.049; the preparation of the *C. acetobutylicum* seed culture is discussed below.

*Clostridium acetobutylicum* spore stock was revived by heating 1 ml at 80°C for 10 min on a heat block before adding heat-shocked spore stock to a 15 ml Falcon tube containing 8 ml of CGM and 0.5 g/ml glucose solution. This culture was subsequently used to make a 1:10, 1:100, and 1:1000 dilution in 3 additional Falcon tubes prepared with 8 ml CGM and 0.5 g/l glucose solution. The spore stock dilution cultures were grown at 39°C in an anaerobic chamber for ∼24 hr until one of the dilutions reached an OD600 of 0.8–1, at which time it could be used to inoculate a seed culture. A total of 1 ml of the 1:100 dilution was used to inoculate a yeast shaker flask containing 37 ml of anaerobic undefined Medium B and 2 ml of maltodextrin at 39°C. The seed culture was placed in the 39°C incubator in the anaerobic chamber to grow for ∼36 hr, until the OD600 reached 0.8–1.0. When the seed culture reached an OD600 of 1.049, four autoclaved yeast shaker flasks containing 0.8 g filter paper, 37 ml anaerobic defined Medium B, and 2 ml 10 wt/vol% maltodextrin at 39°C were inoculated with 1 ml of the *C. acetobutylicum* seed culture to serve as *C. acetobutylicum* monoculture controls.

All cultures were covered with parafilm until the *C. acetobutylicum* inoculum was added to prevent evaporation of the liquid. A sample of 250 μl of Medium B from all experimental replicates was taken before inoculation for later HPLC analysis. Six samples were taken at 2-hr intervals following inoculation the first day of growth. After a 12-hr undisturbed growth period overnight, samples were then taken every 4 hr the second day of growth for a 12-hr period. After another 12-hr undisturbed growth period the second night, samples were again collected every 4 hr for an 8-hr period on the third day of growth, at the end of which time the cultures were harvested, after a total of 56 hr of *C. acetobutylicum* growth. All HPLC samples (250 μl each) were immediately stored in 1.5 ml Eppendorf^™^ tubes at −80°C for later HPLC analysis. These cultures did not reach the OD600 range of the previous cultures, but the cultures were still harvested at this time so that they would still be comparable to the previous co-cultivation experiment's time frame (although the two-stage cultures were allowed to grow for a slightly longer period).

### Long-Term Co-Culture Cultivation (Both One-Stage and Two-Stage)

Prior to *C. churrovis, N. californiae*, or *A. robustus* inoculation, an aliquot of *C. acetobutylicum* spore stock (500 μl) was heat shocked for 10 min at 80°C to revive spores. This aliquot was added to 5 ml MC with 0.5% glucose in a round-bottom 10 ml culture tube and incubated (without shaking) overnight at 39°C in an anaerobic chamber with 100% CO_2_. For the one-stage co-cultivation condition, 50 μl of the *C. acetobutylicum* culture (OD600 = 0.6) was inoculated into a Hungate tube with 10 ml M2 and 0.5 g reed canary grass at the same time as fungal culture passaging and grown for 29 days without shaking. For the two-stage co-cultivation condition, fungi were grown for 22 days without shaking and then *C. acetobutylicum* was inoculated (50 μl of an OD600 = 0.64 overnight culture). The culture was then grown for 10 days without shaking after *C. acetobutylicum* inoculation, with the residual reed canary grass substrate. *C. acetobutylicum* was added to blank M2 as controls using the same methods described for the experimental cultures. Growth of all monocultures and co-cultures were monitored by gas pressure measurements. After each measurement, the gas pressure was vented to 0 psi (Theodorou et al., 1995).

### Harvesting Cultures for RNA Extraction and Sequencing of *C. Acetobutylicum* (Short-Term Two-Stage Condition)

Cultures of *C. acetobutylicum* were grown in the hydrolysate of anaerobic fungus *A. robustus* and harvested after 56 hr of growth as described above. The supernatant was decanted off the filter paper into 50 ml Falcon tubes and the filter paper was discarded. The cell pellet was spun down at 10 000 g and 4°C for 20 min. Visible cell pellets formed for each culture, confirming growth despite low OD measurements. The supernatant was removed from the cell pellet using an automated pipette. A quantity of 500 ul of RNA*later* (Sigma-Aldrich) was added to each cell pellet before storing the samples at −80°C. The samples grown for the one-stage co-cultivation condition were also harvested for RNA extraction, but sequencing failed for those samples.

The cultures were thawed on ice for RNA extraction. The samples were initially spun down in the 50 ml Falcon tubes they had been frozen in at 4°C and 15 000 g for 20 min, but the cells did not form a sufficient pellet in the Falcon tube, so the sample suspended in RNA*later* (Sigma Aldrich) was then transferred by pipetting to a 1.5 ml Eppendorf^™^ tube with a conical bottom and spun down in the microcentrifuge for 3 min at 20 000 RCF and 4°C. The RNA*later* (Sigma Aldrich) was then removed by pipetting from each cell pellet before resuspending in 600 μl buffer RLT. The cell pellet and buffer RLT were then transferred by pipetting to a mortar filled with liquid nitrogen for grinding with a pestle to lyse the cells. After lysis, the samples were processed in the Qiacube using the RNeasy Mini protocol for animal cells with DNAse digest (eluted in 50 ul RNAse-free water). The samples were then stored at −80°C until sequenced.

For transcriptome sequencing, rRNA was removed from 100 ng of total RNA using Qiagen FastSelect 5S/16S/23S for bacterial rRNA depletion (and additional FastSelect plant and/or yeast rRNA depletion) (Qiagen) with RNA blocking oligo technology. The fragmented and rRNA-depleted RNA is reverse transcribed to create first strand cDNA using Illumina TruSeq Stranded mRNA Library prep kit (Illumina) followed by the second strand cDNA synthesis which incorporates dUTP to quench the second strand during amplification. The double stranded cDNA fragments are then A-tailed and ligated to JGI dual indexed Y-adapters, followed by an enrichment of the library by 10 cycles of PCR. The prepared libraries were then quantified using KAPA Illumina library quantification kit (Roche) and run on a LightCycler 480 real-time PCR instrument (Roche). The quantified libraries were then multiplexed and the pool of libraries was then prepared for sequencing on the Illumina NovaSeq 6000 sequencing platform using NovaSeq XP v1 reagent kits (Illumina), S4 flow cell, following a 2 × 150 indexed run recipe. Raw fastq file reads were filtered and trimmed using the JGI QC pipeline resulting in the filtered fastq file. Using BBDuk, raw reads were evaluated for artifact sequence by kmer matching (kmer = 25), allowing one mismatch and detected artifact was trimmed from the 3' end of the reads (*BBDuk: Https://Sourceforge.Net/Projects/Bbmap/*, n.d.). RNA spike-in reads, PhiX reads and reads containing any Ns were removed. Quality trimming was performed using the phred trimming method set at Q6. Finally, following trimming, reads under the length threshold were removed (minimum length 25 bases or 1/3 of the original read length—whichever is longer). Filtered reads from each library were aligned to the *C. acetobutylicum* ATCC 824 reference genome (IMG taxon ID 637000076.fna 1,275,968) using HISAT2 version 2.2.0 (Kim et al., 2015; J. Lee et al., 2008). Strand-specific coverage was generated using deepTools v3.1 (Ramírez et al., 2014). Raw gene counts were generated using featureCounts, with only primary hits assigned to the reverse strand included in the raw gene counts (Liao et al., 2014). Raw gene counts were used to evaluate the level of correlation between biological replicates using Pearson's correlation and determine which replicates would be used in the DGE analysis. One of the four replicates for each condition was removed from the DGE analysis because the Pearson correlation co-efficient fell below .85 for these outliers for at least one other sample in the group. DESeq2 (version 1.28.1) was subsequently used to determine which genes were differentially expressed between pairs of conditions. The parameters used to call a gene DE between conditions were an absolute log_2_fold change ˃1 and a *p*-adjusted value ˂.05. (Love et al., 2014). The average TPM value of either the experimental condition or of the control (or both) for all differentially expressed genes was above a conservative TPM cutoff of 3, indicating biological relevance. Raw gene counts, not normalized counts, are used for DGE analysis, as DESeq2 uses its own internal normalization. Code written to process the data is included in the Supplementary material.

### Detection of Sugars and Fatty Acids by High-Performance Liquid Chromatography 

Levels of volatile fatty acids and sugars present in the supernatant of short-term experimental cultures were measured using an Agilent1260 Infinity HPLC (Agilent). Samples were prepared by acidifying to 5 mM using sulfuric acid and subsequently incubating at room temperature for 5 min. Samples were then centrifuged for 5 min at 21 000 g. The supernatant was syringe filtered into a high-performance liquid chromatography (HPLC) vial (Eppendorf^™^ FA-45–24-11) using a 0.22 μm PVDF filter. Samples were analyzed on an Agilent 1260 Infinity HPLC system (HPLC, Agilent, Santa Clara, CA) equipped with an auto-sampler unit (1260 ALS). Separation was achieved with a Bio-Rad Aminex® HPX-87H Ion Exclusion Column for organic acids (Part No. 1,250,140, Bio-Rad, Hercules, CA) set to 35°C and a flow rate of 0.6 ml/min with a mobile phase of 5 mM sulfuric acid and a 20 μl injection volume. In-house standards were prepared with blank culture medium as a base and sodium formate (ACS Grade, Fisher Chemical S648500), sodium acetate (ACS Grade, Fisher Chemical S210500), l-lactic acid sodium (99%, extra pure, Acros Organics 439 220 100), *n*-butyric acid (99%, Acros Organics, Cat. No. 108 111 000), d-(+)-glucose (Sigma–Aldrich Cat. No. G8270), d-(+)-xylose (Sigma–Aldrich Cat. No. X1500-500 G), 1-butanol, 99.7%, Chromasolv Plus (Sigma–Aldrich Cat. No. 34,867), and ethyl alcohol (molecular biology grade, Sigma–Aldrich Cat. No. E7023) at concentrations of 0.1 and 1–2 g/l (dependent upon the upper concentration limit of the experimental samples).

Quantities of volatile fatty acids and sugars for long-term cultures were measured using an Agilent 1200 equipped with a refractive index detector. After 31 days, cultures were centrifuged at 1286 g for 10 min. The supernatant was filtered through a 0.2 μM sterile filter membrane and a 1 ml aliquot was stored at −20°C until acidification for analysis. The acidification process was the same as that described for the short-term cultures. Separation was achieved with a Bio-Rad Aminex® HPX-87H Ion Exclusion Column for organic acids (Part No. 1 250 140, Bio-Rad, Hercules, CA) set to 65°C and 0.6 ml/min and eluted with a mobile phase of 3.25 mM and 5 mM sulfuric acid and a 20 μl injection volume. Quantification was based on an external calibration curve using pure known components as standards. Stock solutions of 1, 2.5, 5, 10, 25, 50, 100, 250, and 500 mM of pure known components were utilized. Calibration curves were generated by triplicate injections of each calibrator.

## Results and Discussion

**Fig. 1:**
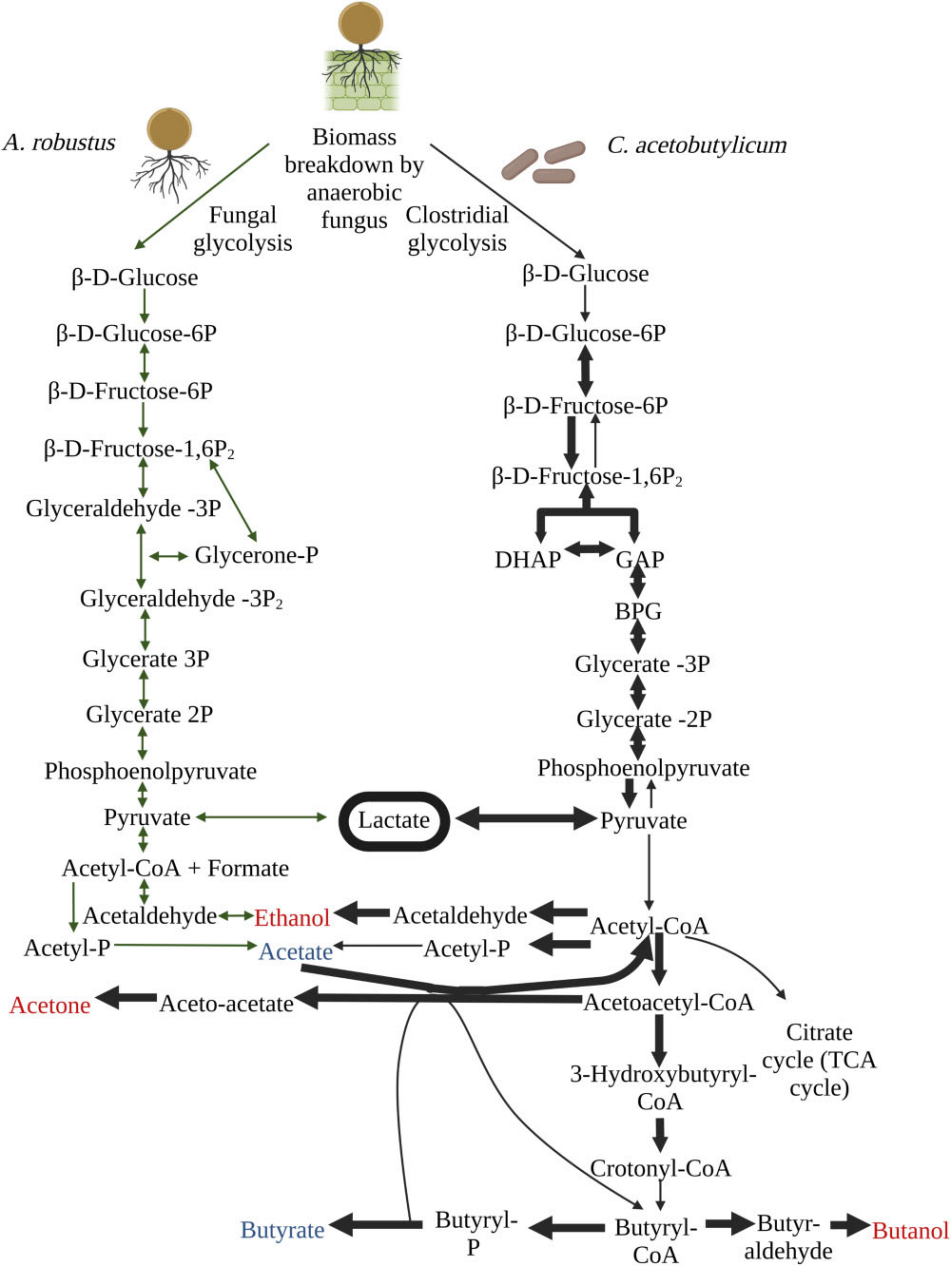
Metabolic map of major fermentation products and potential for lactate cross-feeding in an anaerobic co-culture system composed of anaerobic fungi and anaerobic bacterium *C. acetobutylicum*. The breakdown of plant biomass (lignocellulose or cellulose) is carried out by enzymes secreted by anaerobic fungi, enabling conversion of released glucose by the fungus and bacteria. Major metabolic steps in glucose utilization are shown for the anaerobic fungus *A. robustus* as well as the anaerobic bacterium *C. acetobutylicum*. Red text is used to denote primary products produced by *C. acetobutylicum* under solventogenesis conditions. Blue text denotes primary products produced by *C. acetobutylicum* under acidogenesis conditions. Lactate (circled) is produced by both *C. acetobutylicum* and anaerobic fungi and it is hypothesized that *C. acetobutylicum* can crossfeed lactate via a mechanism for lactate metabolism based on the lactate oxidation pathway in *Acetobacterium woodii*. This mechanism couples a flavin adenine dinucleotide (FAD)-dependent lactate dehydrogenase with an electron flavoprotein complex to convert a reduced ferredoxin, lactate, and two oxidized nicotinamide adenine dinucleotides (NAD) to an oxidized ferredoxin, pyruvate, and two reduced nicotinamide adenine dinucleotides (NADH) (Detman et al., 2019; Schwalm et al., 2019). Bold arrows denote that the TPM count of at least one gene associated with the conversion is equal to or exceeds the median TPM count (491.85) for all genes expressed in the pathways shown via RNA-Seq. TPM counts for individual genes can be found in the Supplementary material. Annotations were obtained from Crown, et al., 2010 and Dash et al., 2014; genes associated with lactate formation were obtained from KEGG (Crown et al., 2010; Dash et al., 2014; Ogata et al., 1999). Genes associated with lactate formation are also in agreement with i802 *C. acetobutylicum* model (Dash et al., 2014). Image made using Biorender.

Anaerobic fungi produce a diverse array of CAZymes capable of converting lignocellulosic biomass to soluble sugars that can support the growth of other microbes (Henske et al., 2018). Here, we leveraged metabolic end products of fungal growth (e.g., fermentable sugars, lactate, acetate, and formate) to enable metabolic cross-feeding to enhance production of industrially-relevant biochemicals through synthetic co-culture. Based on previous work that showed assimilation of the fermentation product lactate by *C. acetobutylicum* (Bahl et al., 1986; Datta & Zeikus, 1985; Diez-Gonzalez et al., 1995), a microbe commonly used in ABE bioprocesses, we hypothesized that partnership between anaerobic fungi and this anaerobic bacterium would elevate bacterial butyrate or butanol production via metabolic cross-feeding of fungal-produced lactate (Fig. 1) (Crown et al., 2010; Dash et al., 2014; Ogata et al., 1999).

**Fig. 2:**
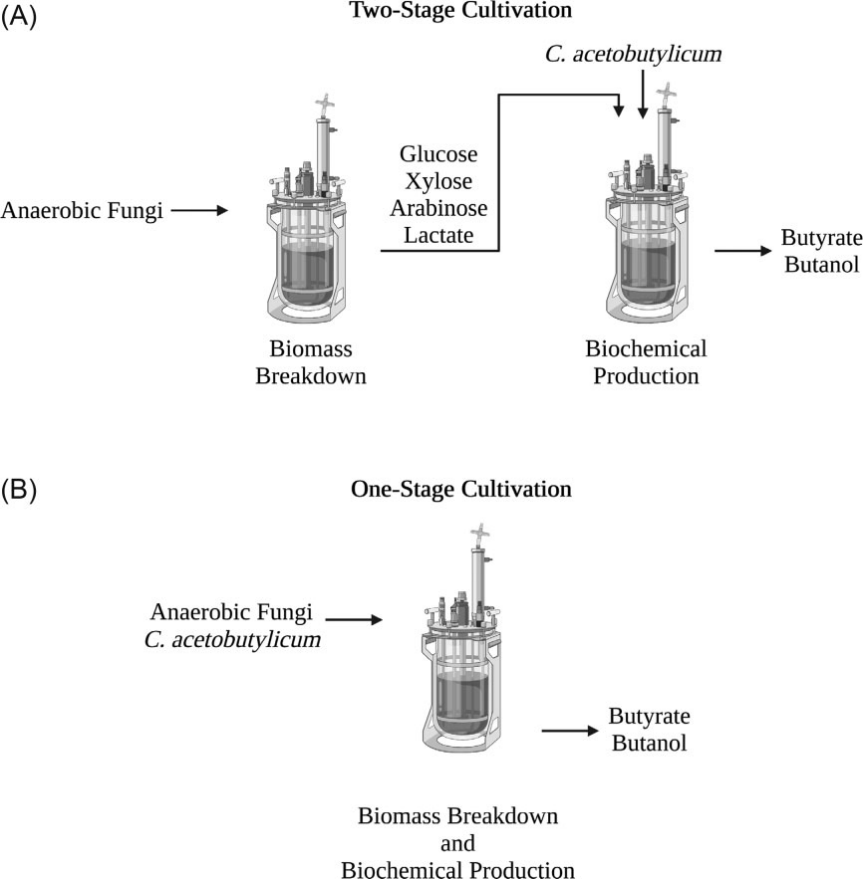
(A) Schematic of the two-stage anaerobic cultivation experiment to produce butanol and butyrate from cellulose or lignocellulose. The anaerobic fungus (either *C. churrovis, N. californiae*, or *A. robustus*) was inoculated into a culture containing reed canary grass and allowed to grow for 22 days. After 22 days of fungal growth, anaerobic bacterium *C. acetobutylicum* was inoculated directly into fungal supernatant with the reed canary grass substrate still remaining in it and grown for 10 days. Analogous short-term experiments were also conducted, whereby anaerobic fungus *A. robustus* was inoculated into a culture containing filter paper and allowed to grow for 8 days to release fermentation products. After 8 days of fungal growth, anaerobic bacterium *C. acetobutylicum* was inoculated directly into sterile-filtered spent fungal cultures and grown for another 56 hr. (B) Schematic of a one-stage simultaneous anaerobic cultivation experiment to produce butanol and butyrate from cellulose or lignocellulose. Anaerobic fungal strains *C. churrovis, A. robustus, or N. californiae* were inoculated into a culture containing reed canary grass as well as *C. acetobutylicum* to facilitate simultaneous metabolic cross-feeding and produce butanol and butyrate over a period of 29 days. Analogous short-term experiments were also conducted, whereby anaerobic fungus *A. robustus* was inoculated into a culture vessel containing filter paper and allowed to grow for 24 hr prior to inoculation with *C. acetobutylicum* for simultaneous growth and release of fermentation products. Image made using Biorender.

To test our hypothesis, we both co-cultivated anaerobic fungi with *C. acetobutylicum* and grew *C. acetobutylicum* in spent fungal supernatant in a defined medium (Fig. 2). The supernatant of two-stage cultures grown in spent fungal supernatant was cultivated for 22 days before *C. acetobutylicum* inoculation and cultures were sampled for fermentation products after 10 days of growth (Fig. 2A). One-stage, or simultaneously co-cultivated, culture supernatant was sampled after 29 days of growth (Fig. 2B). We cultivated the fungus on a complex lignocellulosic substrate, reed canary grass, both in order to better reflect cultivation conditions found in industrial bioprocesses and also because anaerobic fungi are capable of growth on unpretreated lignocellulose. *Clostridium acetobutylicum* was paired with one of three different strains of anaerobic fungus, *Neocallimastix californiae, Anaeromyces, robustus*, or *Caecomyces churrovis* to test whether the metabolic co-cultivation strategy was generalizable across anaerobic fungal strains.

### Gas Production Provides Evidence of Established Anaerobic Co-Cultures

**Fig. 3:**
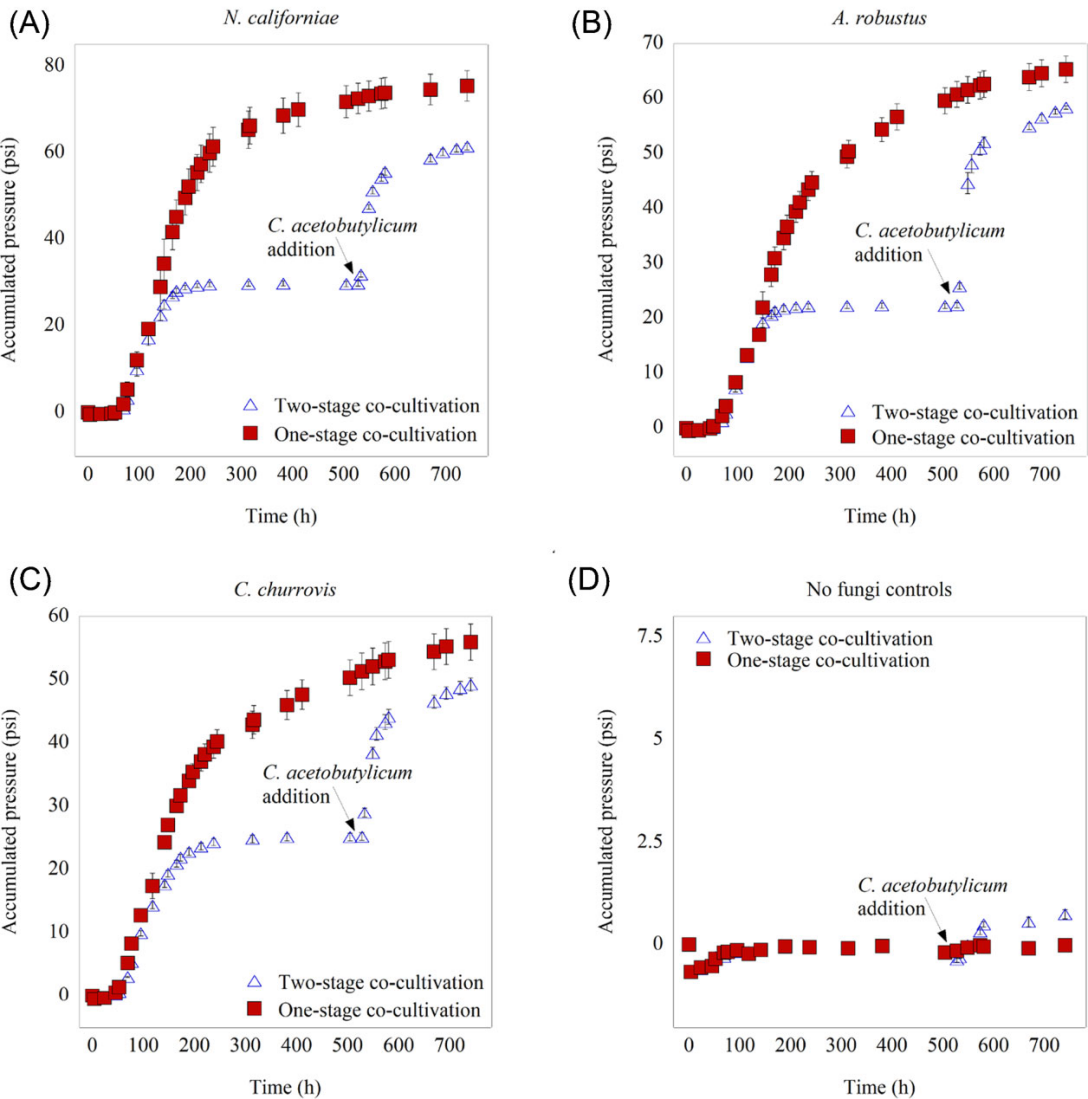
Cumulative pressure data for long-term experiments indicate increased pressure production in the one-stage co-cultivation condition. An arrow is used to indicate when *C. acetobutylicum* was added to cultures for all two-stage conditions. One-stage fermentation with both *C. acetobutylicum* and the indicated fungal strains led to synergistic growth and greater pressure production relative to the two-stage condition for each fungal strain tested. This provides further evidence, in addition to the HPLC data, that suggests that *C. acetobutylicum* cross-feeds lactate from the anaerobic fungi and benefits fungal growth. The mean value is plotted for each set of replicates and error bars indicate standard deviation.

Proliferation of all monocultures and co-cultures was non-invasively monitored by gas pressure production measurements as a proxy for growth, as shown in Fig. 3A–D. In all cases, fermentation gas pressures are well beyond typical values seen for fungal monoculture growth (Henske et al., 2018), indicating that both anaerobic fungi and anaerobic bacteria actively grow in both one-stage and two-stage cultures. For the two-stage growth scheme, *C. acetobutylicum* was quickly able to establish in the culture without an appreciable lag phase and rapidly contributed to fermentation gas production long after cessation of fungal growth as evidenced by the long pressure plateau (Fig. 3A–C). Nevertheless, one-stage fermentation with both *C. acetobutylicum* and the anaerobic fungal strains led to synergistic growth and greater pressure production relative to the two-stage fermentation for all strains tested. Notably, co-cultures that included fungal strains with extensive rhizoidal networks (*A. robustus* and *N. californiae*) (Haitjema et al., 2014) resulted in the highest accumulated gas pressures (Fig. 3A–B) compared to those that contained the non-rhizoid forming fungus *C. churrovis* (Henske et al., 2017) (Fig. 3C). While non-rhizoidal strains such as *C. churrovis* are well-suited for cultivation conditions that allow the concentrations of anaerobic fungi to be measured and subsequent flux calculations to be performed (Leggieri et al., 2021), the current study suggests a potential trade-off to using non-rhizoid-forming fungi to liberate sugar from lignocellulose, which ultimately limits its ability to generate nutrients for a co-cultured partner like *C. acetobutylicum*. Gas pressure readings also showed that addition of *C. acetobutylicum* to reed canary grass in the absence of fungi led to negligible growth, as this bacterium does not possess CAZymes to hydrolyze lignocellulose (Fig. 3D).

### Excess Sugars Released by Anaerobic Fungi Support Growth of *C. acetobutylicum*

**Fig. 4:**
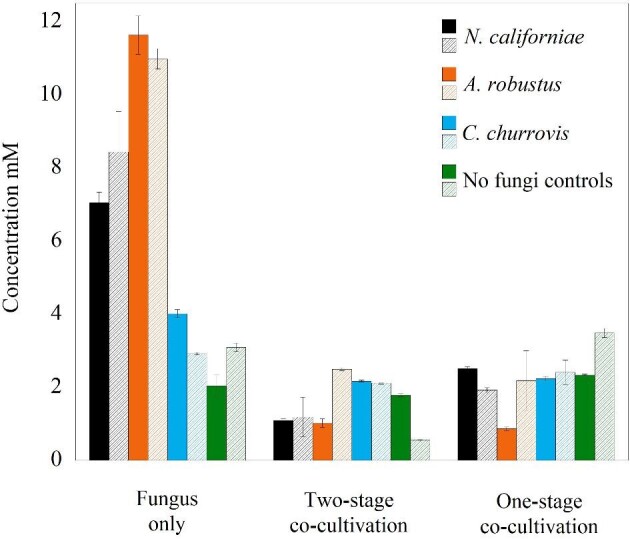
Released fermentable sugars in fungal monocultures versus fungal-bacterial co-cultures grown in M2 on reed canary grass. Sugar concentrations were measured after 29 days of microbial growth for the one-stage co-cultivation condition or 10 days of *C. acetobutylicum* growth for the two-stage co-cultivation condition grown in spent fungal supernatant that the fungi had grown for 22 days previously. Solid fill indicates glucose measured and patterned fill indicates xylose measured. Colors correspond to a particular fungal strain used in the monoculture or co-culture as provided in the legend. Higher levels of glucose and xylose were released compared to sucrose and arabinose (graphs for sucrose and arabinose included in the Supplementary material). With the exception of sucrose, sugars released by the fungi were significantly depleted in all experimental cultures containing *C. acetobutylicum*, indicating that sugars released by the fungus can sustain growth of *C. acetobutylicum*. The mean value is plotted for each set of replicates and error bars indicate standard deviation.

HPLC analysis of spent monoculture and co-culture supernatant provided evidence that lignocellulose-derived sugars supported the growth of *C. acetobutylicum* in co-cultures (Fig. 4). Rhizoidal fungi (*N. californiae* and *A. robustus*) led to the release of 7–11 mM of glucose and 8–11 mM xylose in all experimental conditions, similar to the observation in a previous study that excess sugars released by anaerobic fungi could support the growth of *Saccharomyces cerevisiae* (Henske et al., 2018). In both two-stage and one-stage co-culture conditions, a drastic reduction of fermentable sugars was seen, which is evidence of their assimilation by *C. acetobutylicum*. Sucrose and arabinose were also released by all strains of anaerobic fungi as shown in Fig. S1a and b, but sucrose was not significantly utilized by *C. acetobutylicum* in any experimental condition. While arabinose was consumed by *C. acetobutylicum* in all experimental conditions, the concentration of arabinose released (5–6 mM) was significantly less than the amount of glucose and xylose released by *N. californiae* and *A. robustus*. It has been demonstrated previously that *C. acetobutylicum* consumes arabinose before xylose (Servinsky et al., 2018), however, *C. acetobutylicum* will produce more acetate than butyrate when metabolizing arabinose (Servinsky et al., 2018). The greater increase in butyrate compared to acetate for our data supports the observation that a greater amount of glucose and xylose is consumed compared to arabinose. As compared to the rhizoid-forming fungi, the non-rhizoid forming *C. churrovis* produced a far lower concentration of fermentable sugars (1–2 mM glucose above autoclaved controls). The two-stage cultivation condition has as much or less glucose or xylose as the one-stage cultivation condition for each fungal strain pairing. This is in agreement with the previous observation that less pressure was produced in the two-stage cultivation condition than in the one-stage cultivation condition for all fungal strain pairings as shown in Fig. 3. More sugar would be required in the two-stage cultures if the growth of two-stage cultures eventually exceeded the growth of one-stage cultures. It is reasonable to presume that the fungi are dead or dormant in two-stage cultures, preventing *C. acetobutylicum* from accessing more sugar from the biomass substrate.

### Co-Cultivation Results in Increased Butyrate Production, Lactate Cross-Feeding, and Increased Butanol Production

**Fig. 5:**
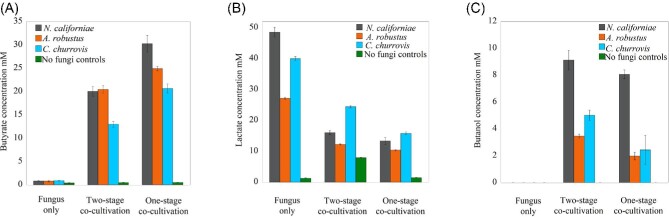
Production of butyrate, lactate, and butanol in cultures grown in M2 on reed canary grass. Concentrations were measured after 29 days of microbial growth for the one-stage co-cultivation condition or 10 days of *C. acetobutylicum* growth in the two-stage co-cultivation condition grown in spent fungal supernatant that the fungi had grown in for 22 days previously. Lactate cross-feeding occurs in both experimental conditions. Butanol was produced in the long-term cultivation condition, in contrast to the short-term cultivation condition. Butyrate levels were significantly increased for all experimental conditions relative to controls. The mean value is plotted for each set of replicates and error bars indicate standard deviation.

In all co-culture conditions tested, significant amounts of both butanol and butyrate were produced from lignocellulose for all co-culture combinations tested (Fig. 5). The amount of butyrate produced by *C. acetobutylicum* one-stage co-cultivation condition was higher than the amount of butyrate produced in the two-stage condition after almost 30 days across all fungal strains tested in the co-culture (Fig. 5A). There was at least 4.5 mM more average butyrate produced in the one-stage cultivation condition versus the two-stage condition for all fungal strains in the study. Conversely, lower levels of lactate were detected in all experimental conditions compared to fungal controls (Fig. 5B), providing evidence that *C. acetobutylicum* likely cross-fed lactate produced by the anaerobic fungi, which bolstered butyrate production (Fig. 5A). The concentrations of acetate and ethanol were found to be slightly to moderately elevated in all co-culture conditions relative to monoculture controls (Fig. S2A and B). Additionally, no significant changes were found in the concentrations of formate in the co-cultures versus monocultures (Fig. S2C), which further supports that lactate assimilation by *C. acetobutylicum* was responsible for elevated butanol and butyrate production. It has been shown previously that clostridium species, *Clostridium beijerinckii, C. propionicum*, and *C. tyrobutyricum* were able to ferment lactate (Bhat & Barker, 1947; Diez-Gonzalez et al., 1995; Woolford, 1984). *Clostridium acetobutylicum* cultures have been shown to metabolize lactate in corn steep liquor and in semi-defined medium with glucose and lactose (Bahl et al., 1986; Datta & Zeikus, 1985). *C. acetobutylicum* strain P262 has been shown to use lactate as an energy source in the presence of acetate as a co-substrate (Diez-Gonzalez et al., 1995).

These cultures also reached the solventogenic growth phase of *C. acetobutylicum*, as demonstrated by the butanol production in all experimental conditions, shown in Fig. 5C. Higher butanol levels were observed in the two-stage experimental condition compared to one-stage co-cultivation for co-cultures formed with the *A. robustus, N. californiae*, and *C. churrovis* strains. These results suggest that two-stage cultivation shifts *C. acetobutylicum* to the solventogenesis phase of growth, which is preferable for production of butanol under these conditions. Conversely, one-stage production under these conditions keeps within the acidogenic growth phase to promote production of more butyrate. This earlier shift to solventogenesis could also explain the lower pressure production in the two-stage condition as shown in Fig. 3.

### Short-Term Two-Stage Cultivation of Anaerobic Fungi and *C. acetobutylicum* also Bolsters Butyrate Production

**Fig. 6:**
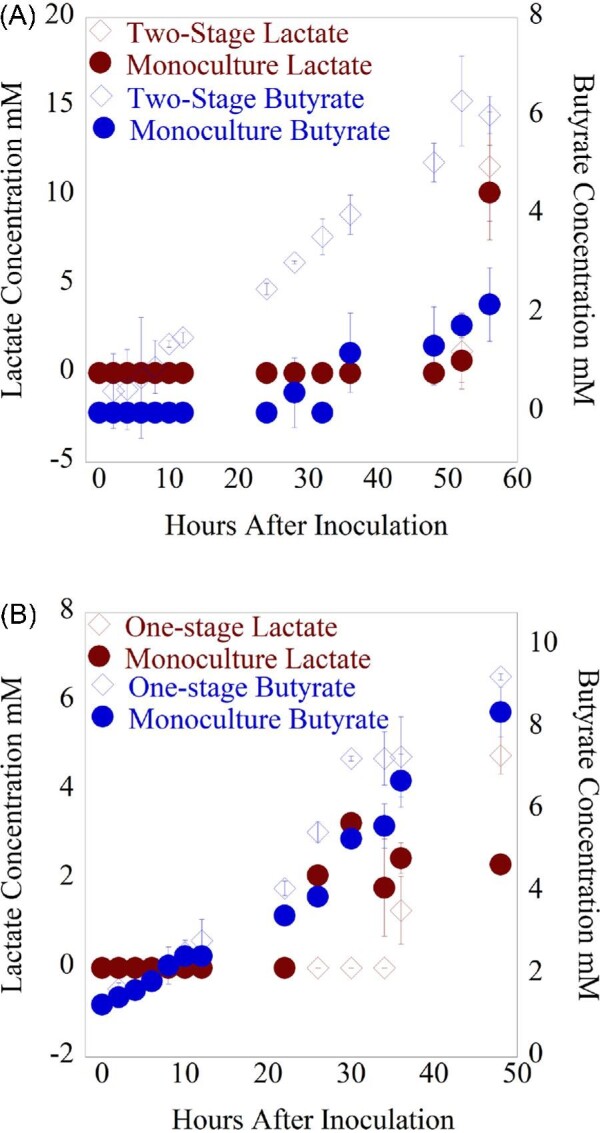
(A) Timecourse graph of lactate and butyrate production for *C. acetobutylicum* cultivated in anaerobic fungal supernatant (short-term co-cultivation) and *C. acetobutylicum* monoculture controls grown in Medium B the fungi had not grown in previously. Sugar release was also measured but did not exceed the limit of detection (0.1 g/l). (B) Timecourse graphs (short-term co-cultivation) of lactate and butyrate production for *C. acetobutylicum* co-cultured with the anaerobic fungal strain *A. robustus* and *C. acetobutylicum* monoculture controls. Sugars released were also measured but did not exceed the limit of detection in any of the cultures (0.1 g/l). Significantly higher levels of lactate were detected in the cultures in which *C. acetobutylicum* was co-cultivated with actively growing *A. robustus*, even though fungal monoculture controls did not grow enough to produce fungal metabolite levels above the limit of detection. The mean value is plotted for each set of replicates and error bars indicate standard deviation.

We also both simultaneously co-cultivated the anaerobic fungus *A. robustus* and *C. acetobutylicum* and grew *C. acetobutylicum* in spent *A. robustus* fungal supernatant in a defined medium short-term (see methods) to examine any metabolic and/or transcriptional response of *C. acetobutylicum* to fungal co-cultivation. While fungal metabolites and released sugars did not accumulate in an amount that exceeded the limit of detection for HPLC readings in the duration of the experiment (eight days of growth), a significant increase in butyrate production was observed in *C. acetobutylicum* cultures grown in spent fungal medium vs. *C. acetobutylicum* controls grown in the defined medium, as shown in Fig. 6A. These cultures were kept in the anaerobic chamber in loosely capped culture vessels so gas production could not be measured. OD600 levels were measured upon harvest but were comparable for both conditions. At the final timepoint upon harvest at 56 hr, over 2.5 times more butyrate was measured in the cultures of *C. acetobutylicum* grown in spent fungal supernatant versus the *C. acetobutylicum* controls grown in the defined medium. Production of lactate, acetate, and ethanol did not significantly differ between experimental and control conditions as shown in Fig. 6A and Fig. S3, although the amount of lactate measured in the culture at 55 hr after inoculation (when the cultures were harvested for RNA extraction) significantly increased in both experimental and control cultures while the amount of measured ethanol in the cultures dropped, indicating a metabolic switch to the conversion of pyruvate to lactate instead of acetyl-CoA.

For the two-stage co-culture with *A. robustus*, we transcriptionally verified the metabolic pathways of *C. acetobutylicum*, including core carbon metabolism genes (glycolysis, acetate, butyrate, butanol, and acetone fermentative pathways) as shown in Fig. 1. Both *C. acetobutylicum* and *A. robustus* produce lactate and acetate. Ethanol is produced by both microbes when *C. acetobutylicum* enters the solventogenic phase of metabolism. Transcripts per million (TPM) counts indicated that genes associated with core carbon metabolism were actively expressed. The observed metabolic switch to the conversion of pyruvate to lactate instead of acetyl-CoA at 56 hours after inoculation (when the cultures were harvested for RNA extraction) was also reflected in TPM counts. TPM counts for genes associated with the conversion of pyruvate to lactate exceeded the median TPM count for all genes in core carbon metabolism pathways, while TPM counts for genes associated with the conversion of pyruvate to acetyl-CoA fell below the median TPM count (shown in Fig. 1). Total TPM counts for annotated genes are included in Supplementary material.

As mentioned previously, 2.5 times more butyrate was produced in cultures of *C. acetobutylicum* grown in spent fungal supernatant from *A. robustus* cultures than in cultures grown in Medium B controls without fungal supernatant. Overall, the high expression of the gene (CA_P0035) that produces the enzyme acetaldehyde dehydrogenase (EC 1.2.1.10)/alcohol dehydrogenase AdhE (EC 1.1.1.1) is in agreement with the high levels of ethanol production relative to acetate. The lack of butanol would also indicate that this enzyme is associated with the conversion of acetyl-CoA to ethanol as opposed to the conversion of butyryl-CoA to butanol. Butyryl-CoA is metabolically downstream of acetyl CoA, thus the shunting of acetyl-CoA to ethanol is a possible explanation for the limited butanol production (Yoo et al., 2015).

### RNA-Seq Indicates that Two-Stage Cultivation May Decrease Time Required to Reach Solventogenesis and to Relieve Carbon Catabolite Repression

Differential gene expression analysis of *C. acetobutylicum* in the short-term two-stage cultivation condition with *A. robustus* hydrolysate vs. the *C. acetobutylicum* monoculture condition revealed that 94 genes were upregulated in the two-stage condition and 64 genes were upregulated in the monoculture condition. Lists of upregulated genes are provided in Supplementary material. Three of the top ten genes upregulated in the two-stage condition are associated with solventogenic pathways (Table 1). Genes CA_P0163 and CA_P0164 encode the alpha and beta subunits of butyrate-acetoacetate CoA-transferase (*ctfA* and *ctfB*) associated with the conversion of acetoacetyl-CoA to acetoacetate, indicating that metabolism in the two-stage condition is likely further along in solventogenesis compared to the monoculture. The alcohol dehydrogenase *adhE1* (CA_P0162), which catalyzes butanol production, was also upregulated in the two-stage condition, further supporting that *C. acetobutylicum* was further into solventogenesis compared to monoculture. It is well-established that these pSOL1 megaplasmid genes are expressed at the onset of solventogenesis (Harris et al., 2002; Sauer & Dürre, 1995) and were shown to be transcriptionally upregulated in a previous study at the onset of solvent formation (Alsaker & Papoutsakis, 2005). In a study by Alsaker et al., other pSOL1 megaplasmid and chromosomal genes (CA_P0165, CA_C3299, and CA_C3298) were upregulated at the onset of solvent formation, and expression increased continuously throughout stationary phase with the exception of CA_C3299 (Alsaker & Papoutsakis, 2005). In our study, CA_P0165 was also upregulated in the two-stage condition at a log2fold change of 1.63 (not reaching the cutoff for the top 10 most highly upregulated genes). Since butanol was not detected in the cultures upon harvest and pSOL1 megaplasmid genes CA_C3299 and CA_C3298 were not yet upregulated, it is possible that the switch to solventogenesis was just beginning at the time of harvest. Though the monoculture displayed reduced expression of the solvent genes mentioned above, CA_C0017 which encodes a seryl-tRNA synthetase was upregulated in the monoculture and has been shown previously to be upregulated during solventogenesis (Yoo et al., 2015). These results may indicate that *C. acetobutylicum* reaches the solventogenesis phase faster in the two-stage experimental culture condition compared to monoculture.

**Table 1.** Top ten upregulated genes in *C. acetobutylicum* in the two-stage co-cultivation condition versus *C. acetobutylicum* monoculture. Genes highlighted in yellow are associated with metabolic pathways involved in metabolite production for *C. acetobutylicum*. Genes highlighted in blue are associated with cellulose degradation, although *C. acetobutylicum* has not been shown to degrade cellulose.

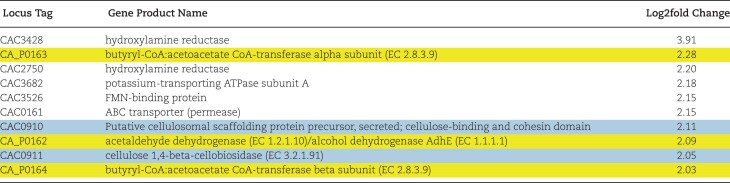

Of the genes upregulated in the monoculture condition, CA_C3037 which encodes the carbon catabolite repressor *ccpA* was particularly interesting (Supplementary material). This finding corresponded to several predicted or experimentally confirmed (Ren et al., 2012) CcpA regulated genes that were upregulated in the two-stage condition, including genes encoding a potassium-transporting ATPase (CA_C3682), glucanase (CA_C2807), pentose utilization enzyme (CA_C1349), and pectate lyase (CA_C1968). These upregulated genes indicate that the two-stage condition resulted in relieved carbon catabolite repression at the time of sampling.

Although it does not degrade lignocellulose, the *C. acetobutylicum* genome does contain several cellulase-encoding genes and a complete cellulosome cluster of genes, and a low level of induction of cellulase activity has been shown to occur during growth on xylose (López-Contreras et al., 2004). Genes CA_C0910 and CA_C0911, both highlighted in blue in Table 1, are associated with cellulose-binding and cellulose degradation (Nolling et al., 2001). Notably, these genes have not been found to be regulated by CcpA (Ren et al., 2012); however, CA_C0911 has been shown to have decreased protein abundance for *C. acetobutylicum* grown on glucose compared to xylose, thus indicating potential carbon catabolite mediated regulation (López-Contreras et al., 2004). In addition to the two genes associated with cellulose degradation found in the top 10 upregulated genes list, 5 more are upregulated with a log2foldchange ˃1, for a total of 7 out of the 12 genes associated with cellulose degradation (Nolling et al., 2001) being upregulated in the two-stage cultivation condition, shown in Table S1. One of the genes associated with cellulose-binding and cellulose degradation (*celF*, CA_C0911), a family 48 glycoside hydrolase enzyme with exoglucanase activity, is a conserved feature between cellulosomes produced by clostridial species and appears to contribute to cellulosome function (López-Contreras et al., 2004).

### Short-Term One-Stage Cultivation Results in Increased Lactate Production

In our short-term study, butyrate production in the co-cultivation experimental condition did not significantly differ between the co-cultivation and monoculture control conditions (Fig. 6B). However, twice the lactate was produced in the co-cultivation condition versus the *C. acetobutylicum* monoculture control (Fig. 6B). This could be due to lactate production by the fungus, although lactate levels in fungal monoculture controls did not exceed the limit of detection during the experiment; therefore, it is also possible that the fungus was instead supporting increased lactate production by *C. acetobutylicum* through the release of sugars from the cellulose substrate. Acetate and ethanol levels were slightly higher in the co-cultivation conditions, as shown in Fig. S4, again due to either metabolite production by the anaerobic fungus or due to increased clostridial growth due to the release of sugars. No butanol production was observed in either of the short-term cultivation conditions, indicating that the cultures did not enter the solventogenic growth phase in the duration of these experiments, making it unlikely that the observed increase in ethanol in the single stage co-cultivation condition was due to increased clostridial growth. The drop in ethanol and corresponding increase in lactate was not observed for the one-stage co-cultivation condition, but this could be due to a slightly earlier harvest time in an effort to obtain high-quality fungal RNA from the cultures. However, the lactate flux through the anaerobic fungi and *C. acetobutylicum* could be high despite the low steady-state concentration; resolving this flux would require transcriptomic sequencing of the mixed culture.

## Conclusion

Higher levels of butyrate and butanol in long-term fungal and *C. acetobutylicum* cultures reveal that creating consortia that include these two microbes could be a promising future avenue of industrial bio-butyrate and biobutanol production from lignocellulosic feedstocks, specifically. Evidence is presented in this study that strongly suggests lactate cross-feeding between anaerobic fungi and *C. acetobutylicum*. This cross-feeding is likely a cause of the observed increase in butyrate production in the experimental co-cultivation conditions explored in this study. Additional experiments are needed to definitively pinpoint this metabolic exchange, particularly carbon tracing experiments since lactate is produced by both *C. acetobutylicum* and anaerobic fungi. RNA extraction methods also need to be developed to examine the late-stage cultures and reliably sequence mixtures of prokaryotic and eukaryotic organisms in co-culture. Future studies could include co-cultivating anaerobic fungi and *C. acetobutylicum* to convert non-homogenous lignocellulose feedstock to produce tunable outputs. As demonstrated in this study, fungal strains vary in their capacity to release excess sugars from lignocellulosic biomass, so other fungal strains not used in this work could yield superior results in a similar co-culture. Combining anaerobic fungi with methanogens to potentially enhance lignocellulose degradation could increase the amount of excess sugars released to support the growth of *C. acetobutylicum* (Li et al., 2022). As demonstrated by a previous study that combined *C. acetobutylicum* with undefined rumen fluid to hydrolyze pretreated agave and enhance hydrogen and butanol production (Morales-Martínez et al., 2020), varying experimental conditions such as temperature and pH could further enhance biobutanol production. It may be possible to tune products or induce early solventogenesis by supplementing the medium, which would keep the fermentation pH high and prolong fungal growth (M. Mukherjee et al., 2019; Rao & Mutharasan, 1987; S. Wang et al., 2012).

## Supplementary Material

kuac024_Supplemental_Files

## Data Availability

RNA-Seq data for *C. acetobutylicum* monocultures and *C. acetobutylicum* cultures grown in *A. robustus* fungal supernatant has been deposited under the BioProject accession number PRJNA831884. GOLD was used for the metadata curation and facilitating NCBI submissions from the Joint Genome Institute (S. Mukherjee et al., 2021).
