# Supplementary material for: Co‑cultivation of anaerobic fungi with Clostridium acetobutylicum bolsters butyrate and butanol production from cellulose and lignocellulose
Source: J Ind Microbiol Biotechnol. 2022 Nov 3;49(6):kuac024. doi: 10.1093/jimb/kuac024 (PMC9923384; doi:10.1093/jimb/kuac024)
Supplement: kuac024_Supplemental_Files [file kuac024_supplemental_files.zip › Brown_JIMB_Supplement_formatted.docx]

**Supplementary Information**

| A | |  | | | | | | | | | B |  | | | | | | |  |  |  |  |  |  |
| --- | --- | --- | --- | --- | --- | --- | --- | --- | --- | --- | --- | --- | --- | --- | --- | --- | --- | --- | --- | --- | --- | --- | --- | --- |
|  | | 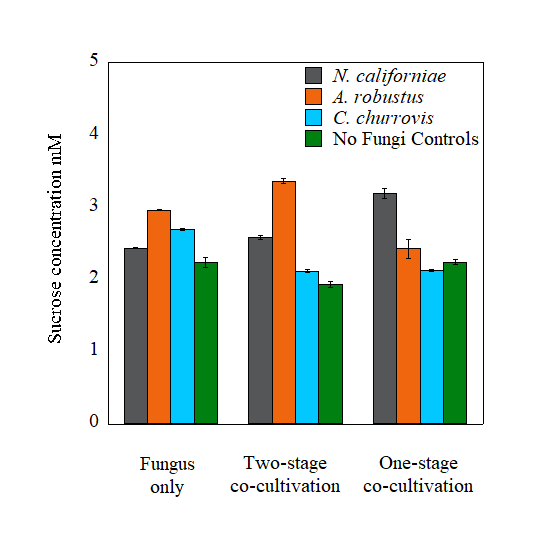 | | | | | | | | |  | 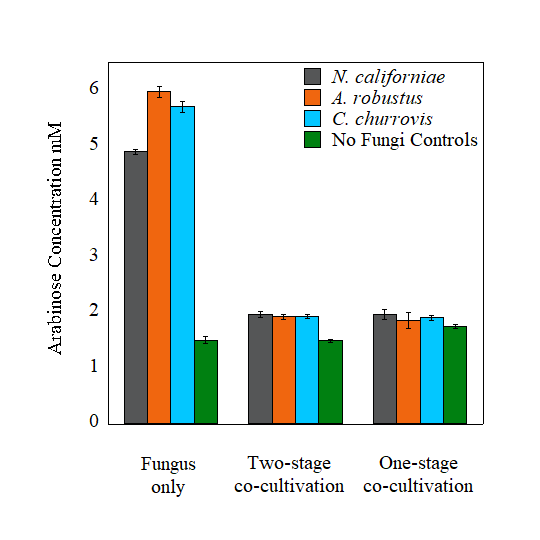 | | | | | | |  |  |  |  |  |  |
| **Supplementary Figure 1**. Sucrose and arabinose sugars in cultures grown on M2 in reed canary grass long-term. With the exception of sucrose, sugars released by the fungi were significantly depleted in all experimental cultures containing *C. acetobutylicum*, indicating that sugars released by the fungus can sustain growth of *C. acetobutylicum*. | | | | | | | | | | | | | | | | | |  |  |  |  |  |  |  |
| A |  | | | | | | | | B | | | | | | | | | | | | | | | |
|  |  | |  | | | | |  | | | | | | | | | | | | |  |  |  |  |
|  | 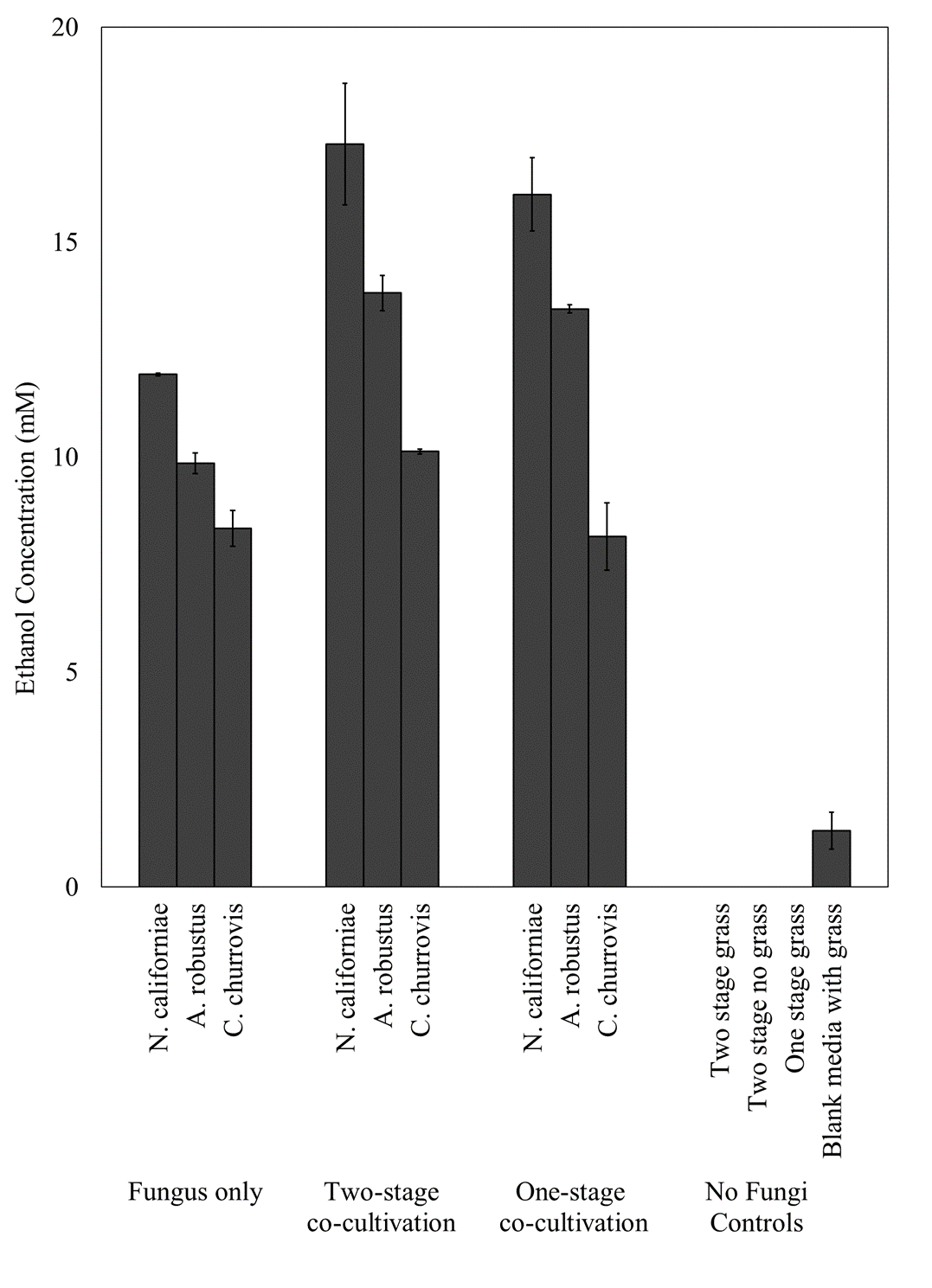 | | | | | | | |  | | 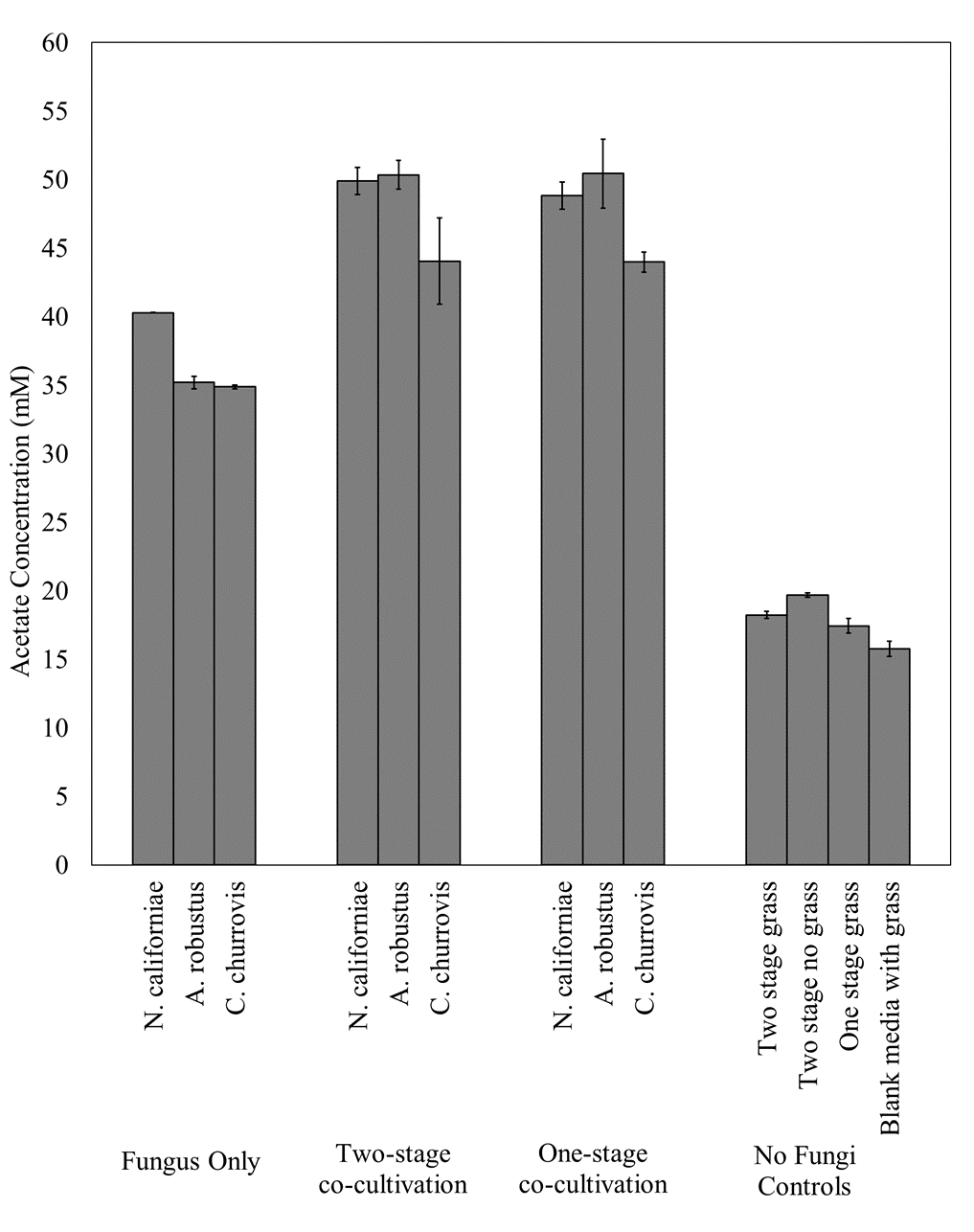 | | | | | | | | | | | | |  |
|  | C | | | | | | | | | | | | | | | |  | | | | | | | |
|  | 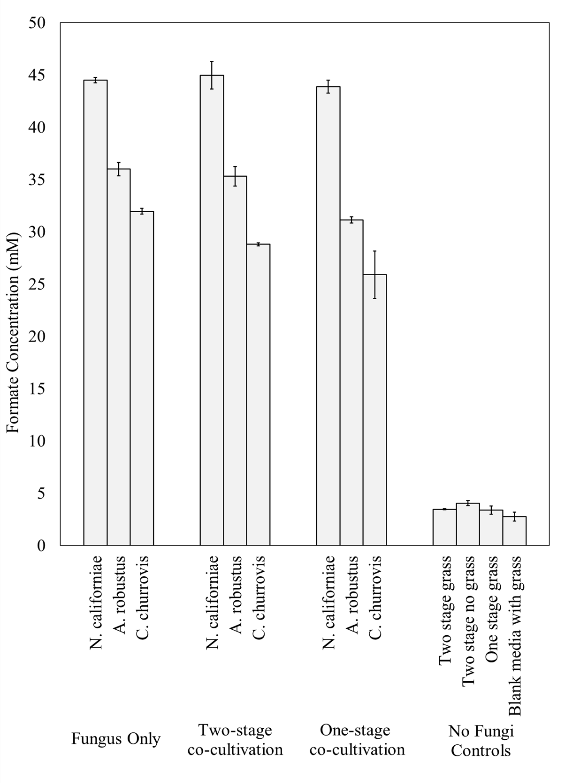 | | | | | | | | | | | | | | | |  | | | | | | | |
|  |  | | | | | |  |  |  |  |  |  |  |  |  |  |  |  |  |  |  |  |  |  |
| **Supplementary Figure 2**. Production of the fatty acids ethanol, acetate, and formate in cultures grown in M2 with reed canary grass after 29 days of microbial growth for the one-stage co-cultivation condition or 10 days of *C. acetobutylicum* growth in the two-stage conditions grown in spent fungal supernatant that the fungi had grown for 22 days previously. Levels of these metabolites did not vary significantly between the two-stage and one-stage cultivation conditions for any fungal strain used in the study. | | | | | | | | | | | | | | | | | | | | | | |  |  |
| A | | |  | | | | | B | |  | | | | | | |  |  |  |  |  |  |  |  |
|  |  | |  |  |  |  |  |  |  |  |  |  |  |  |  |  |  |  |  |  |  |  |  |  |
|  | 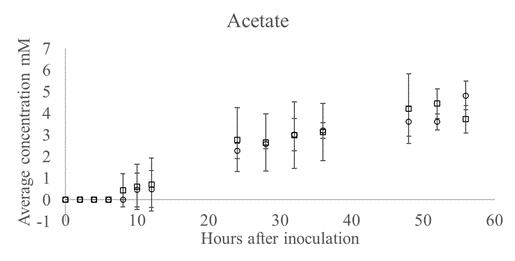 | | | | | | | | | | | | 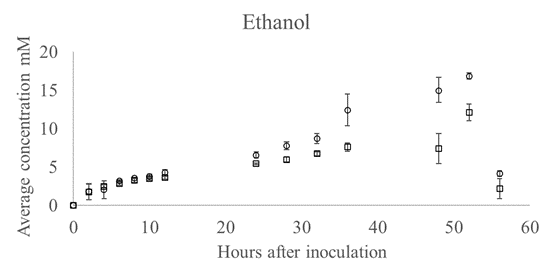 | | | | | | |  |  |  |  |  |
|  | C | | | | | | | |  | | | | |  | | | |  |  |  |  |  |  |  |
|  | 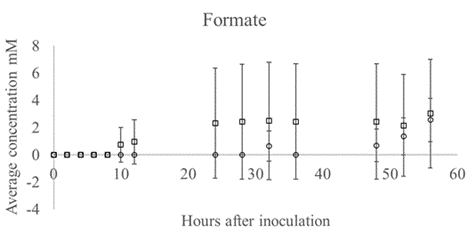 | | | | | | | | | | | | | | | |  |  |  |  |  |  |  |  |
|  |  | | | | | | | | | | | | | | | |  |  |  |  |  |  |  |  |
| **Supplementary Figure 3.** Timecourse graphs of HPLC readings of acetate, ethanol, and formate production for *C. acetobutylicum* cultivated in anaerobic fungal supernatant (two-stage cultivation condition) and *C. acetobutylicum* controls grown in media the fungi had not grown in previously. | | | | | | | | | | | | | | | | | | | | | | |  |  |

**Supplementary Table 1**. Upregulated *C. acetobutylicum* genes associated with cellulose degradation in the two-stage cultivation condition versus *C. acetobutylicum* monoculture. Differential gene expression analysis indicated that 7 out of the 12 genes associated with cellulose degradation were upregulated in the two-stage cultivation condition compared to *C. acetobutylicum* controls. None of the 12 genes associated with cellulose degradation were downregulated.

| **Locus Tag** | **Gene Product Name** | **Log2fold Change** |
| --- | --- | --- |
| CAC0911 | cellulose 1,4-beta-cellobiosidase (EC 3.2.1.91) | 2.05 |
|  |  |  |
| CAC0912 | Possible non-processive endoglucanase family 5, secreted; CelA homolog secreted; dockerin domain | 1.93 |
|  |  |  |
| CAC0913 | endoglucanase Cel9G | 1.77 |
|  |  |  |
| CAC0916 | endoglucanase (EC:3.2.1.4) | 1.14 |
|  |  |  |
| CAC0561 | non-processive endocellulase | 1.81 |
|  |  |  |
| CAC0915 | Endoglucanase A precursor (endo-1,4-beta-glucanase) (cellulase A), secreted; dockerin domain | 1.19 |
|  |  |  |
| CAC0910 | Probably cellulosomal scaffolding protein precursor, secreted; cellulose-binding and cohesin domain | 2.11 |

|  | 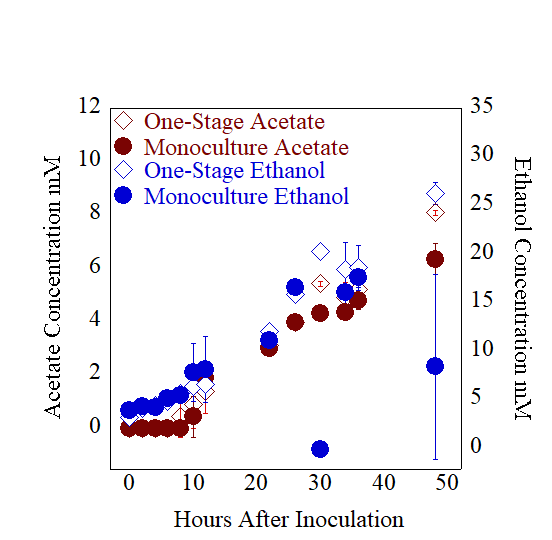 |
| --- | --- |
|  | |
| **Supplementary Figure 4**. Timecourse graph of HPLC readings of acetate and ethanol production for *C. acetobutylicum* co-cultured with the anaerobic fungal strain *A. robustus* (one-stage cultivation condition) and *C. acetobutylicum* monoculture controls. | |
